# Treatment of chronic HCV genotype 1 infection with telaprevir: a Bayesian mixed treatment comparison of fixed-length and response-guided treatment regimens in treatment-naïve and –experienced patients

**DOI:** 10.1186/1471-230X-13-148

**Published:** 2013-10-14

**Authors:** Armin D Goralczyk, Silke Cameron, Ahmad Amanzada

**Affiliations:** 1 Division of Gastroenterology and Endocrinology, University Medical Center Goettingen, Georg-August-University Goettingen, Goettingen, Germany; 2 Division of Gastroenterology and Endocrinology, University Medical Center, Robert-Koch-Straße 40, Goettingen, 37075, Germany

**Keywords:** Chronic HCV infection, Protease inhibitor, Telaprevir, Bayesian mixed treatment comparison

## Abstract

**Background:**

Telaprevir (TVR) has been approved for response-guided-therapy (RGT) of chronic hepatitis C (HCV) genotype-1-infection in treatment-naïve and –experienced patients. In RGT-regimens patients that did not achieve extended rapid-virological-response (eRVR) within the first 4–12 weeks undergo treatment for 48-weeks, whereas in fixed-length-treatment (FLT) patients are treated for a fixed-duration regardless of their RVR.

**Methods:**

This systematic review and Bayesian mixed-treatment-comparison (MTC) aimed to compare the efficacy and safety of standard-therapy with pegylated-interferon-α/ribavirin (Peg-IFN-α/RBV (48 weeks), group A), FLT with TVR, Peg-IFN-α/RBV for 12 weeks with a long (+36 weeks, group B) or short (+12 weeks, group C) tail of Peg-IFN-α/RBV treatment, and RGT with 12 weeks of TVR, Peg-IFN-α/RBV followed by 12 weeks of Peg-IFN-α/RBV (group D) or no therapy (group E).

**Results:**

We identified seven randomized controlled trials including 3505 patients. Compared to standard-treatment (group A), treatment-naïve patients allocated to groups B, C, and D were significantly more likely to achieve sustained-virological-response (SVR, odds ratios (OR): B vs. A 3.5 (credibility interval [CrI] 2.2-5.4), C vs. A 3.0 (CrI 1.8-4.9), D vs. A 3.4 (CrI 2.5-4.6)). Treatment-experienced patients achieved increased SVR rates when they were treated in group B (OR: 8.2 (CrI 5.0-13.5)), C (OR 7.0 (CrI 3.9-12.8)), or simulated group D (OR 8.2 (CrI 4.3-15.3)). Patients treated with short RGT (simulated group E) did also have a significant improvement when they were treatment-experienced (simulated OR 3.6 (CrI 1.6-8.2)), whereas the effect was not significant in treatment-naïve patients (OR E vs. A 1.6 (CrI 0.9-2.7)).

**Conclusion:**

Long FLT and RGT regimens are useful treatment options for HCV-genotype-1 in both treatment-naïve and -experienced patients. A short 24-weeks FLT regimen does not seem to be inferior and should further be evaluated in clinical trials to reduce side effects and costs of treatment.

## Background

Approximately 170 million people worldwide are infected chronically with the hepatitis C virus (HCV) [1]. Since 2001, the combination of pegylated interferon-α (Peg-IFN-α) plus ribavirin (RBV) has been the standard-of-care treatment for patients with HCV infection [2-4]. New direct-acting antiviral agents such as HCV NS3/4A protease inhibitors are one of the most promising drug targets for HCV [5]. NS3/4A HCV protease inhibitors achieve high antiviral potency by blocking HCV polyprotein cleavage and may also neutralize HCV NS3 protease-mediated interference with the innate immune system. Through this mechanism, HCV NS3/4A protease inhibitors reverse the HCV NS3 protein’s capacity to block intracellular signal-transduction pathways for endogenous IFN production in vitro and may also do so in vivo [6,7]. The recent approval of two novel HCV nonstructural protein NS3/4A protease inhibitors (telaprevir and boceprevir) heralds a new era in the treatment of chronic hepatitis C [8-13]. Clinical trials demonstrate that telaprevir (TVR) in combination with Peg-IFN-α and RBV improve treatment efficacy in both treatment-naïve [10,14-16] and -experienced patients [11,13] when compared to the standard treatment of Peg-IFN-α and RBV. These large randomized trials examined different treatment regimens: Fixed-length treatment (FLT) versus response-guided treatment (RGT) duration as a triple therapy including TVR with a varying Peg-IFN-α and RBV treatment tail. TVR has to be administered three times per day with a fatty meal. Treatment with TVR is more expensive and also has additional side effects including rash, pruritus, anemia, dysgeusia and gastrointestinal symptoms. The rate of treatment discontinuation in patients treated with a TVR-based therapy was between 10-18% [11,13,17]. The Food and Drug administration (FDA) approved TVR as RGT for treatment-naïve and prior Peg-IFN-α and RBV relapsers without prospective evaluations in randomized controlled trials [18]. Because of the higher rate of adverse effects and costs associated with these various therapeutic options referring to the use of TVR, it is important to determine the relative efficacy and safety of these FLT and RGT options using TVR as compared to that of the standard treatment consisting of Peg-IFN-α and RBV only.

## Methods

The inclusion and exclusion criteria, outcome measures, methods of literature search, selection, data extraction and statistical analysis were defined in a protocol according to the recommendations in the Cochrane Handbook for Systematic Reviews of Interventions [19].

### Endpoints and definitions

The primary endpoint was the proportion of patients who achieved sustained virological response (SVR) defined as undetectable plasma HCV RNA 24 weeks after the last planned dose of the study treatment. The secondary end points were safety, as assessed by the percentage of patients who discontinued therapy due to adverse events (AE) and the incidence of serious adverse events (SAE) as defined by the International Conference on Harmonisation, Guidelines for Good Clinical Practice (ICH GCP) [20].

Undetectable HCV-RNA at week 4 of treatment was defined as rapid virological response (RVR). When RVR was persistent in the following weeks it was defined as extended RVR (eRVR). Relapse occurs when HCV RNA decreases and remains below the limit of detection during treatment but becomes detectable after cessation of treatment. Null-response was defined as less than < 2 log_10_ reduction in serum HCV RNA from baseline during treatment. Partial response was defined as ≥ 2 log_10_ reduction from baseline HCV RNA at week 12, but with virus remaining detectable through week 24 or end of treatment.

### Inclusion and exclusion criteria

For inclusion into this analysis, studies had to fulfill the following criteria. They had to be: (1) randomized, prospective trials; (2) compare the efficacy of TVR with conventional doses of Peg-IFN-α-2a (180 μg/week) or Peg-IFN-α-2b (1.5 μg/kg of body weight/week), both in combination with RBV; (3) report the primary outcomes, i.e., SVR, as defined above; and had to be performed (4) in patients with a chronic HCV genotype 1 infection.

**Figure 1:**
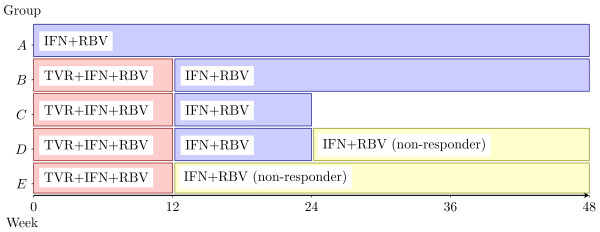
Treatment regimens; TVR: telaprevir; IFN: pegylated interferon-α; RBV: ribavirin.

Additionally, included trials had to report results on at least two of the following five treatment groups (Figure 1): (A) standard treatment with Peg-IFN-α and RBV for 48 weeks; (B) FLT with either 12 weeks of TVR in combination with Peg-IFN-α and RBV followed by 36 weeks of treatment with Peg-IFN-α and RBV or 24 weeks of treatment with TVR, Peg-IFN-α, and RBV followed by 24 weeks of Peg-IFN-α and RBV (overall treatment duration 48 weeks); (C) 12 weeks of TVR in combination with Peg-IFN-α and RBV followed by 12 weeks of treatment with Peg-IFN-α and RBV (overall treatment duration 24 weeks); (D) RGT with 12 weeks of TVR, Peg-IFN-α and RBV followed by 12 weeks of treatment with Peg-IFN-α and RBV for patients that had eRVR and 36 weeks of treatment for patients that had no eRVR; (E) RGT with 12 weeks of TVR, Peg-IFN-α and RBV followed by no further treatment for patients that had eRVR or 36 weeks of treatment with Peg-IFN-α and RBV for patients that had no eRVR. Treatment arms in which patients received no RBV or a treatment duration with TVR that was less than 12 weeks duration were excluded.

Only recently researchers affiliated with the FDA published an analysis of Peg-INF-α, RBV and TVR regimens who demonstrated that interferon responsiveness does not change in Peg-IFN-α/RBV-experienced subjects with a second round of Peg-IFN-α/RBV [18,21]. Based on this assumption we included both studies with treatment-naïve and -experienced patients, i.e. patients that already had treatment with Peg-IFN-α/RBV but had relapse, null or partial response. To account for differences in baseline responsiveness and magnitude of the effect of TVR we stratified the statistical analysis to account for treatment status, i.e., treatment-experienced versus treatment-naïve.

### Literature search, selection, and data extraction

A systematic literature search was performed without language restrictions from inception to 25 February 2013 in the following databases: Medline/PubMed and Web of Science. The keywords used were “telaprevir” or “VX-950”. General reviews, meta-analyses, and references from published randomized controlled trials (RCTs) and presentation to the International Association for the Study of the Liver, the European Association for the Study of the Liver and the American Association for the Study of Liver Diseases Meetings were also searched for additional citations. Publications were reviewed independently by two reviewers (A.A. and A.D.G) in a two-stage process. Disagreement and any discrepancies were resolved by discussion. Data extraction was performed by both reviewers, using a standardized form.

### Risk of bias

The risk of bias was assessed independently by both reviewers (A.A. and A.D.G.) without blinding to journal and authorship. The quality items assessed were sequence generation, allocation concealment, blinding of participants and outcome, completeness of follow-up, and whether problems with incomplete outcome data were appropriately addressed. We also assessed selective reporting and a defined low risk of bias when a detailed study registration was available and the complete study protocol had been published. Assessments were performed according to the definitions stated in the Cochrane Handbook [19]. We included data on all randomized patients, regardless of treatment completion. Data concerning only those patients who completed the assigned treatment (“per protocol”) was not available for most included studies and could therefore not be evaluated.

### Data analysis

Bayesian mixed treatment comparison (MTC) meta-analysis is increasingly used when there are multiple possible treatment options that have not been compared directly but each has been compared to a standard treatment, or when two or more new drugs have been tested against a common standard but no direct evidence on a head-to-head comparison is available. The framework of Bayesian MTC meta-analysis as described by Lu et al. [22] combining direct and indirect evidence on any comparison of the defined treatment groups was utilized. This method of MTC allows an indirect comparison of treatments A and B, although only data on studies comparing one of the two treatments against a third comparator C can be analyzed. For example, TVR and boceprevir were compared in a MTC meta-analysis [23], and the indirect evidence suggests that TVR may be more efficacious for prior relapse but not for treatment naïve patients.

The model utilized included random effect baselines as well as homogenous treatment variance. The model was extended to account for different study populations, i.e., treatment-naïve and -experienced patients, by stratification. In general, we used vague priors and calculated median and 95% credibility intervals (CrI), which are the Bayesian equivalent of confidence intervals from the posterior distribution. Analyses were performed with *R* version 2.15.2 [24] in combination with *JAGS*[25]. Additionally, we used the *R* packages *R2jags, ggplot2*, and *ggmcmc* to analyze and visualize results of the Markov Chain Monte Carlo (MCMC) simulation.

In all MCMC analyses, acceptable convergence was reached as indicated by the visual analysis of the traceplots, the running mean and the density of the posterior distribution of the separate chains (all provided as supplemental material). The Gelman and Rubin statistic for all parameters presented was below 1.05. Because of autocorrelation for some parameters, we applied thinning of the chains and reached acceptable effective sample sizes for all analyzed parameters.

## Results

**Figure 2:**
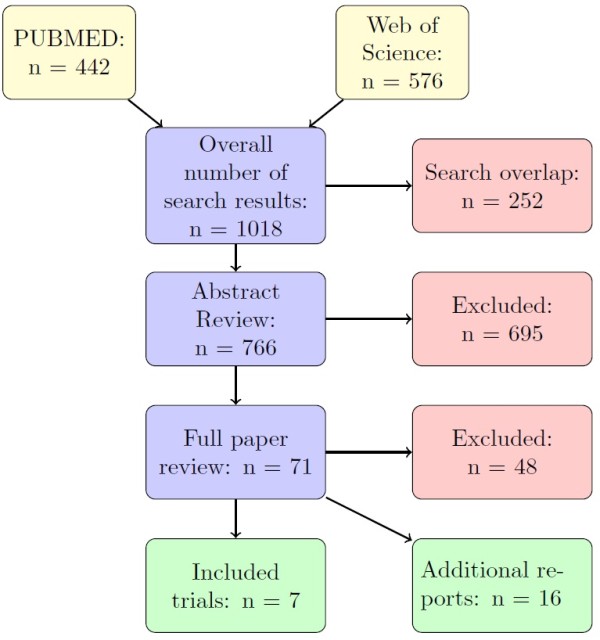
Flow chart of systematic review of telaprevir.

A total of 1018 publications were identified through electronic database and manual search using the above reported keywords (Figure 2), of which 252 were excluded as duplicates. Of the 766 publications that qualified for abstract review, 695 were excluded primarily because they were not randomized or TVR was not compared in the study. 71 were considered potentially relevant but only 7 [10,11,13-17] met the inclusion criteria and were included in the meta-analysis.

Table 1 shows the characteristics of the included studies and allocation of their treatment arms to the defined treatment groups of this review. Seven published phase II and III randomized placebo-controlled trials provided efficacy and safety data among 3505 patients treated with TVR in combination with Peg-IFN-α plus RBV. Five of these trials were conducted in 2390 treatment-naïve patients [10,15-17] and two were conducted in 1115 treatment-experienced patients [11,13]. The populations were comparable in terms of age and gender. In terms of genosubtype, the study of Kumada et al. [15] and Hezode et al. [16] represented an exception since a population was included that predominantly contained genosubtype 1b; genosubtype 1a was the most common genotype among the populations recruited in all other trials. Of note, none of these trials allowed the use of erythropoietin therapy to treat anemia.

**Table 1:** Characteristics of included trials

**Trial**	**Treatment experience**	**Treatment type**	**Overall treatment duration (weeks) #**	**Treatment group**	**No of patients**	**Age (median)**	**Male sex no (%)**	**HCV genotype 1b no (%)**	**TVR treatment (weeks)**	**Peg-IFN-α dose (weeks)**	**RBV dose (mg/d)**
[16] McHutchison et al. 2009	Naïve	RGT	12	E	17	49	12 (71)	6 (35)	12	α2a: 180 μg	1000-1200
		RGT	24	D	79	49	54 (68)	17 (22)	12	α2a: 180 μg	1000-1200
		FLT	48	B	79	50	48 (61)	27 (34)	12	α2a: 180 μg	1000-1200
		Control	48	A	75	49	43 (57)	20 (27)	none	α2a: 180 μg	1000-1200
[14] Hezode et al. 2009	Naïve	RGT	12	E	82	44	49 (60)	45 (55)	12	α2a: 180 μg	1000-1200
		RGT	24	D	81	46	54 (67)	50 (62)	12	α2a: 180 μg	1000-1200
		RGT	12	NI	78	45	43 (55)	38 (49)	12	α2a: 180 μg	none
		Control	48	A	82	45	46 (56)	45 (55)	none	α2a: 180 μg	1000-1200
[11] McHutchison et al. 2010	Experienced	FLT	24	C	115	51	78 (68)	33 (29)	12	α2a: 180 μg	1000-1200
		FLT	48	B	113	52	80 (71)	42 (37)	24	α2a: 180 μg	1000-1200
		FLT	24	NI	111	53	72 (65)	36 (32)	24	α2a: 180 μg	none
		Control	48	A	114	50	76 (67)	34 (30)	none	α2a: 180 μg	1000-1200
[10] Jacobson et al. 2011	Naïve	RGT	24 (+24)	D	363	49	214 (59)	149 (41)	12	α2a: 180 μg	1000-1200
		RGT	24 (+24)	NI	364	49	211 (58)	151 (41)	8	α2a: 180 μg	1000-1200
		Control	48	A	361	49	211 (58)	151 (42)	none	α2a: 180 μg	1000-1200
[13] Zeuzem et al.	Experienced	FLT	48	B	266	51	183 (69)	121 (45)	12	α2a: 180 μg	1000-1200
		FLT	48	NI	264	51	189 (72)	115 (44)	16	α2a: 180 μg	1000-1200
		Control	48	A	132	50	88 (67)	59 (45)	none	α2a: 180 μg	1000-1200
[17] Sherman et al. 2011	Naïve	RGT	24 (+24)	D	221	NA	NA	NA	12	α2a: 180 μg	1000-1200
		FLT	48	B	219	NA	NA	NA	12	α2a: 180 μg	1000-1200
[15] Kumada et al. 2012	Naïve	FLT	24	C	126	53	66 (52)	124 (98)	12	α2b: 1.5 μg/kg	600-1000
		Control	48	A	63	55	33 (52)	63 (100)	none	α2b: 1.5 μg/kg	600-1000

No: number; TVR: telaprevir; mg: milligram; d: day; RBV: ribavirin; RGT: response-guided treatment; FLT: fixed-length treatment; control: standard treatment; NI: not included; NA: not available; *pseudo-groups merged from original dosing; # in RGT-regimens patients that did not achieve extended rapid-virological-response (eRVR) within the first 4–12 weeks undergo treatment of overall 48-weeks.

The overall treatment duration varied between 12 to 48 weeks with a TVR treatment duration of 12 weeks. Two treatment arms included different TVR-treatment durations, one with 8 weeks [10] and the other with 24 weeks [11] (Table 1) as well as two arms without the use of RBV [11,14]. Those were excluded from the analysis. Two trials [10,16] were designed to report on what seemed to be FLT, however patients in the TVR-treatment arms that did not have eRVR were considered non-responders and were continued on Peg-IFN-α/RBV for an overall treatment length of 48 weeks. These trials could not be included in the analysis as FLT regimens as it was unclear, how many patients without eRVR would eventually reach SVR without extending the treatment. In both trials, the authors report SVR data after extension of the treatment to the full 48 weeks course. These data were included in our analysis as RGT regimens. In the trial of Sherman et al. [17], all patients received 12 weeks of triple therapy. Patients without eRVR continued on Peg-IFN-α/RBV for a full course of 48 weeks whereas patients with eRVR were randomized to receive only 12 or 36 weeks of treatment with Peg-IFN-α/RBV. From these three cohorts we simulated two cohorts with a 48 weeks FLT regimen and a 24+24 weeks RGT treatment.

TVR was given as a single dose of 1250 or 1125 mg on study day 1, followed by a dose of 750 mg every 8 hours orally in the 3 phase 2 trials [11,16]. It was administered orally at a dose of 750 mg every 8 hours with food in the 4 phase 3 trials [10,13,15,17]. All the included trials utilized Peg-IFN-α2a at 180 μg/week and a weight adjusted RBV dose between 1000 to 1200 mg/day except the trial published by Kumada et al. [15] who used Peg-IFN-α2b at 1.5 μg/kg/week and a weight adjusted RBV dose between 600 to 1000 mg/d (Table 1).

All trials were randomized and all but the ILLUMINATE trial published by Sherman and colleagues were double blind. Six studies [10,11,13,16,17] were funded by pharmaceutical companies. The methods of trial conduct and statistical analysis were adequate and documented in detail in the original publication or the study protocol, which was published as supplemental material. Only insufficient information concerning the risk of bias was available for the study published by Kumada and colleagues [15].

Three studies measured the plasma HCV-RNA level with the use of the COBAS TaqMan HCV assay, version 1.0 (Roche Molecular Systems), with a lower limit of quantification of 30 IU per milliliter and a lower limit of detection of 10 IU per milliliter [11,14,16]. Four studies measured the plasma HCV-RNA level with the use of the COBAS TaqMan assay (Roche Molecular Systems, version 2.0), which has a lower limit of quantification of 25 IU per milliliter and a lower limit of detection of approximately 10 to 15 IU per milliliter [10,13,15,17].

**Figure 3:**
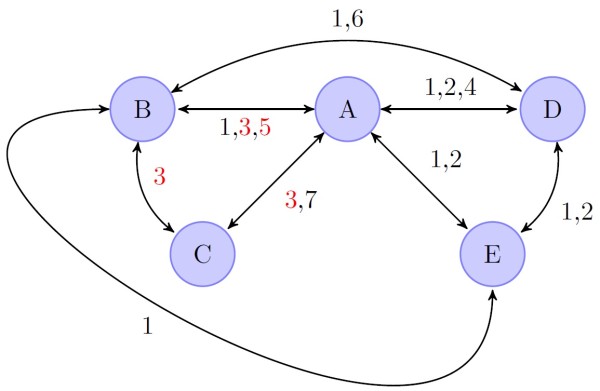
**Structure of mixed treatment comparison network.** Node labels: **A**, standard treatment (Peg-IFN-α/RBV 48 weeks); **B**, fixed-length treatment (FLT) with long tail (TVR 12 weeks/Peg-IFN-α/RBV 48 weeks); **C**, FLT with short tail (TVR 12 weeks/ Peg-IFN-α/RBV 24 weeks); **D**, response-guided treatment (RGT) with tail (TVR 12 weeks/ Peg-IFN-α/RBV 24 weeks/or Peg-IFN-α/RBV 24 weeks); **E**, RGT without tail (TVR 12 weeks/ Peg-IFN-α/RBV 12 or 36 weeks). Edges indicate comparisons in trials denoted by number (see Table 1); black numbers: treatment-naïve patients; red numbers: treatment experienced patients.

The structure of the MTC network is shown in Figure 3 with the naming scheme of the nodes corresponding to the treatment regimens as shown in Figure 1. Several trials compared more than two treatment arms [10,11,13,16].

It must be noted that only two studies [11,13] were performed with treatment-experienced patients, which only provided data for regimen groups A, B and C. Hence, treatment-experienced patients have only been studied in FLT regimens although the FDA has approved RGT treatment for prior relapsers [18].

### Efficacy in treatment-naïve patients

**Figure 4:**
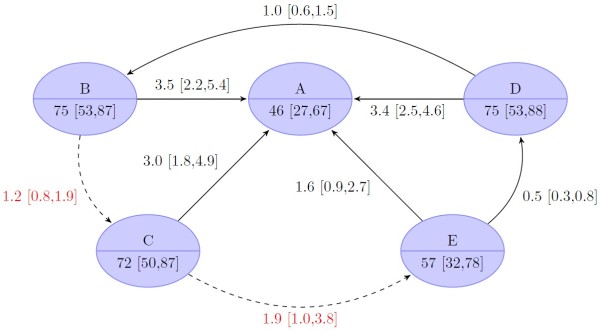
**Results of bayesian mixed treatment comparison for treatment-naïve patients.** The upper node part indicates the treatment group [Node labels: **A**, standard treatment (Peg-IFN-α/RBV 48 weeks); **B**, fixed-length treatment (FLT) with long tail (TVR 12/24 weeks/Peg-IFN-α/RBV 48 weeks); **C**, FLT with short tail (TVR 12 weeks/Peg-IFN-α/RBV 24 weeks); **D**, response-guided treatment (RGT) with tail (TVR 12 weeks/Peg-IFN-α/RBV 24 and/or 24 weeks); **E**, RGT tail (TVR 12 weeks/Peg-IFN-α/RBV 12 or 36 weeks), the lower node part gives the median proportion of patients (in percent) with sustained virological response (SVR, credible 95% bayesian intervals in square brackets). The edges indicate comparisons with the arrowhead indicating the comparator (i.e. baseline). The odds ratio of the respective comparison is shown with credible 95-% intervals of the bayesian analysis in square brackets. Dashed lines (and red text color) indicate comparison that was not observed but only simulated.

The primary efficacy endpoint (Figure 4) SVR, was reached significantly more often in the two FLT TVR regimens (75% [CrI 53 to 87%] in group B, 72% [CrI 50 to 87%] in group C) and in the long RGT triple regimen (group D: 75% [CrI 53 to 88%]) compared to standard treatment (group A: 46% [CrI 27 to 67%]). There was no significant difference for the short RGT TVR regimen (group E: 57% [CrI 32 to 78%]) compared to the standard treatment. Comparison of the TVR regimens shows that there was no significant difference between the two FLT regimens and the long RGT treatment. All three were found to be superior to the short RGT regimen. This result implies that the long RGT regimen is not inferior to a 48 weeks FLT regimen (OR of group D vs. B: 1 [CrI 0.6 to 1.5]). Furthermore, the long RGT regimen and the 48 weeks FLT regimen were not superior to the 24 weeks FLT (OR of group B vs. C 1.2 [CrI 0.8 to 1.9] and D vs. C 1.2 [CrI 0.7 to 1.9]).

### Efficacy in treatment-experienced patients

**Figure 5:**
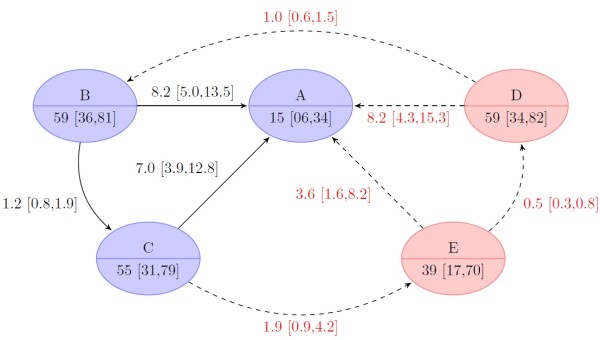
**Results of bayesian mixed treatment comparison for treatment experienced patients.** The upper node part indicates the treatment group [Node labels: **A**, standard treatment (Peg-IFN-α/RBV 48 weeks); **B**, fixed-length treatment (FLT) with long tail (TVR 12/24 weeks/Peg-IFN-α/RBV 48 weeks); **C**, FLT with short tail (TVR 12 weeks/Peg-IFN-α/RBV 24 weeks); **D**, response-guided treatment (RGT) with tail (TVR 12 weeks/Peg-IFN-α/RBV 24 and/or 24 weeks); **E**, RGT without tail (TVR 12 weeks/Peg-IFN-α/RBV 12 or 36 weeks), the lower node part gives the median proportion of patients (in percent) with sustained virological response (SVR, credible 95% bayesian intervals in square brackets). Nodes with red background have not been observed and have only been simulated from the bayesian model. The edges indicate comparisons with the arrowhead indicating the comparator (i.e. baseline). The odds ratio of the respective comparison is shown with credible 95-% intervals of the bayesian analysis in square brackets. Dashed lines (and red text color) indicate comparison that was not observed but only simulated.

Only two pivotal studies were performed for treatment-experienced patients. They provided data only for regimens of group A, B and C. Group D and E were simulated in this bayesian MTC from direct and indirect evidence obtained in treatment-naïve and -experienced patients. As expected, overall treatment efficacy was lower in treatment- experienced patients (Figure 5). The rate of SVR was significantly higher in each of the four TVR regimens – 59% (CrI 36 to 81%) in group B, 55% (CrI 31 to 79%) in group C, 59% (CrI 34 to 82%) in the simulated group D, and 39% (CrI 17 to 70) in the simulated group E – compared to standard treatment (group A: 15% [CrI 6 to 34%]). The results of patients treated with the short FLT regimen were comparable to the results for the long FLT and simulated long RGT regimen. In contrast, the simulated short RGT regimen was inferior as compared to the simulated long RGT regimen.

### Safety of triple therapy

**Figure 6:**
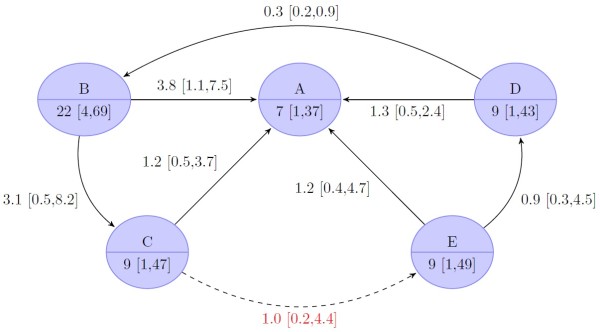
**Serious adverse events of mixed treatment comparison network.** Node labels: **A**, standard treatment (Peg-IFN-α/RBV 48 weeks); **B**, fixed-length treatment (FLT) with long tail (TVR 12/24 weeks/Peg-IFN-α/RBV 48 weeks); **C**, FLT with short tail (TVR 12 weeks/Peg-IFN-α/RBV 24 weeks); **D**, response-guided treatment (RGT) with tail (TVR 12 weeks/Peg-IFN-α/RBV 24 and/or 24 weeks); **E**, RGT without tail (TVR 12 weeks/Peg-IFN-α/RBV 12 or 36 weeks). The edges indicate comparisons with the arrowhead indicating the comparator (i.e. baseline). The odds ratio of the respective comparison is shown with credible 95-% intervals of the bayesian analysis in square brackets. Dashed lines (and red text color) indicate comparison that was not observed but only simulated.

**Figure 7:**
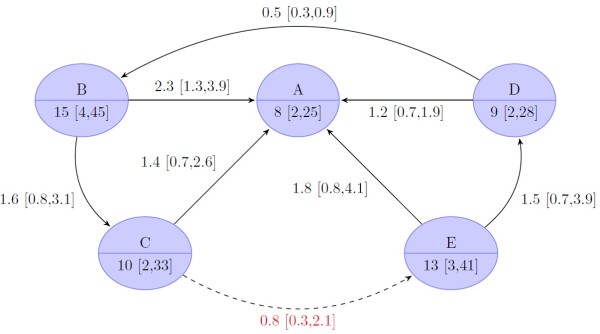
**Serious adverse events leading to treatment discontinuation. A**, standard treatment (Peg-IFN-α/RBV 48 weeks); **B**, fixed-length treatment (FLT) with long tail (TVR 12/24 weeks/Peg-IFN-α/RBV 48 weeks); **C**, FLT with short tail (TVR 12 weeks/Peg-IFN-α/RBV 24 weeks); **D**, response-guided treatment (RGT) with tail (TVR 12 weeks/Peg-IFN-α/RBV 24 and/or 24 weeks); **E**, RGT without tail (TVR 12 weeks/Peg-IFN-α/RBV 12 or 36 weeks).

The expected incidences of SAE and AE leading to treatment discontinuation are shown in Figures 6 and 7. Both, SAE and AE leading to treatment discontinuation were observed significantly more often in treatment group B compared to the control group (OR 3.8 [CrI 1.1 to 7.5] and 2.3 [CrI 1.3 to 3.9], respectively), whereas there was only a small albeit non-significant increase in SAE/AE in the other treatment groups. SAE/AE in the RGT groups D and E and in the short FLT group C were lower than in the long-tail treatment group B, e.g., for group D vs. B the OR for SAE was 0.5 (CrI 0.3 to 0.9) and the OR for AE leading to discontinuation was 0.3 (CrI 0.2 to 0.9). Between treatment groups C through E there was no significant difference in the incidence of SAE and AE leading to treatment discontinuation.

## Discussion

This study compared the efficacy and safety of different TVR-based triple therapy regimens in treatment-naïve and -experienced chronic HCV genotype 1 infected patients. Although these different treatment regimens still have not been yet compared in a randomized trial but triple therapy with TVR has been approved for treatment of HCV genotype 1 infections. Utilizing both direct and indirect evidence, various treatment groups and a network of comparisons (Figure 3) were used for a Bayesian MTC-analysis to estimate the effect of TVR-based regimens compared not only to a common comparator, i.e., standard therapy, but also to each other.

The main results of this analysis are that (i) the addition of TVR increases the likelihood of achieving SVR in treatment-naïve and -experienced chronic HCV genotype 1 patients, (ii) the long FLT and RGT regimen (groups B and D) are equally effective, (iii) the short RGT regimen (group E) is only marginally more effective compared to standard therapy and it is inferior compared to the other regimens, (iv) the short FLT regimen (group C) is non-inferior to the long FLT and RGT regimens. Off note is that the results of group D and E for treatment-experienced patients presented in this study was simulated and has not been applied in prospective randomized studies yet.

Several large multi-center trials have been performed to assess the efficacy and safety of TVR in patients with chronic HCV genotype 1 infection. The results show that in most patients and under most circumstances, TVR increases the likelihood of achieving a SVR. Different TVR treatment strategies were employed to document the superiority of TVR but also to explore differing treatment durations. As expected, not all regimens were equally effective. Hofmann et al. [26] conclude that RGT with 12 weeks of TVR, Peg-IFN-α and RBV, followed by 12 weeks of Peg-IFN-α and RBV for all patients and an additional 24 weeks of Peg-IFN-α and RBV for patients who did not achieve RVR appears to be the best treatment regimen, although no direct comparison to other treatment options, e.g., short FLT TVR-therapy exists, and it has not been assessed in treatment experienced patients.

In our analysis, TVR-based treatment regimen demonstrated a boost in achieving SVR in both treatment-naïve and –experienced patients, with the OR value in treatment-naïve patients lower than in treatment-experienced patients. Moreover, the FLT regimen showed better overall SVR rates and OR results in treatment-naïve patients compared to the RGT regimen. Patients with a FLT duration of 12 weeks triple therapy followed by 12 weeks of Peg-IFN-α and RBV in total showed a SVR rate of 72% despite a shorter treatment duration. The length of treatment duration in RGT in TVR protocols is being discussed. In one open-label study designed to investigate the effects of RGT, patients with eRVR to triple therapy were randomized to 24 or 48 weeks of standard treatment to evaluate whether 48-week therapy can gain further benefits [17]. The results showed no further benefit from the 12-week triple therapy over the 48-week standard therapy in patients with eRVR. Collectively, the TVR 12 and Peg-IFN-α/RBV 24 weeks protocol resulted to be more effective and could shorten the therapy duration compared with standard treatment, thus possibly serving as the treatment of choice for chronic HCV genotype 1 infection. Interestingly, the patients of group E did not achieve significantly higher SVR rates compared to a standard treatment for 48 weeks. Although SVR rates may not be statistically different, it should be acknowledged that the 12 weeks triple regimen is much shorter than the 48 weeks standard treatment and may therefore offer reduction of side effects at equal efficacy. Our data suggest that the chance of treatment failure is likely to be similar despite a longer treatment duration, and that FLT regimen would be a good option if the virus is cleared early. It may be possible to prospectively study this hypothesis in prior partial and null responders; however, the study size and enrollment time for such a trial is likely to be impractical given the rapid pace of HCV drug development. This issue is now a point of interest as the FDA approved a RGT scheme for treatment-naïve and –experienced patients [18]. At the time when the FDA approved this regimen, only three phase 3 studies had evaluated the RGT regimen [10,13,17]. The RGT regimen was prospectively evaluated only in two registration trials of treatment-naïve subjects [10,17]. In these studies, the RGT regimen allowed subjects who achieved eRVR, to stop all treatments at week 24. The RGT regimen was not prospectively evaluated in prior relapsers. Therefore, the FDA used data from cross-study comparison which indicated that SVR rates were 62-77% in prior partial responders and 62-71% in prior null responders who achieved eRVR, irrespective of standard treatment duration (24 or 48 weeks), suggesting that the RGT regimen might also be suitable in prior partial and null responders [18]. Our simulated data for treatment-experienced patients shows that a RGT regimen for 12 weeks triple therapy followed by either 12 or 24 weeks of standard treatment could achieve a SVR of 59% with an OR of 8.2 compared to the control group.

Safety is the biggest concern when TVR-based therapy regimens are used. Despite theencouraging efficacy in terms of enhanced SVR and decreased relapse rates, TVR combination treatment is coupled with an increased incidence of adverse events, including but not limited to rash, anemia, pruritus, anemia, fatigue, flu-like syndrome, headache, nausea, insomnia, diarrhea, and pyrexia, among which rash and anemia are the most common adverse events. These adverse events can lead to discontinuation of treatment in severe cases. The incidences of both SAE and discontinuation of treatment are increased with TVR-based regimen, as shown in this Bayesian MTC. Therefore, the use of TVR is limited in interferon-intolerant patients, and caution should be taken when TVR is used over a long period of time. Since the currently used standard therapy already has numerous adverse effects, we tried to discover whether the potentially shortened treatment duration by adding TVR can reduce the drug toxicity of a longer treatment period with Peg-IFN-α and RBV. Interestingly, this Bayesian MTC demonstrated that the group of patients that initially received 12 weeks triple therapy followed by a fixed-length 24-weeks-treatment with Peg-IFN-α and RBV had the lowest rate of adverse events and discontinuation rate.

### Strength and weaknesses

In this analysis the results from treatment-naïve or –experienced patients were pooled and analyzed. Five of the included trials investigated the efficacy and safety of TVR in treatment-naïve patients, where only two studies examined treatment-experienced patients. This is a major limitation of this Bayesian MTC. Recently, empirical cross-trial data on triple therapy presented and analyzed by the FDA [18] implied that in prior relapsers, interferon responsiveness does not change in Peg-IFN-α/RBV-experienced subjects. The rationale to pool different patient populations was based on this report. Because the difference in previous treatment status may increase heterogeneity and become a potential liability, in this Bayesian MTC a stratified analysis was performed. The stratified MTC analysis allows, for the first time, an estimate of the effect of RGT in treatment-experienced patients to be determined. Nevertheless, it must be noted that the results of treatment-experienced patients with RGT regimen are only simulated.

Another considerable difference in the patient population was the definition of in- or exclusion criteria for patients with cirrhosis, as it is known to be associated with a reduced SVR [2,4]. In two trials patients with cirrhosis were excluded [16], whereas in the others, only patients with decompensated cirrhosis were excluded. A stratified analysis was not possible due to the small number of trials that excluded patients with cirrhosis. Differences in efficacy of TVR in cirrhosis were therefore not analyzed.

There are seven randomized trials included in this Bayesian MTC. Six studies were supported by pharmaceutical industry, which may weaken the strength of evidence demonstrated in this article, although the number of patients included in these 7 randomized trails was fairly large. The heterogeneity of the study collective for this analysis is relatively high, due to the small number of included randomized trails and the different study populations and treatment durations. Inter-study variation may compromise the reliability of these results. However, sensitivity analysis showed that the results of our analysis were stable and reliable. Finally, most of the populations studied here are caucasians, thus the conclusion may not be true for other races or areas. All these limitations point out the direction for future studies.

The therapy options for individuals with chronic HCV genotype 1 infection are widening. To offer these patients the best possible therapy, various predictive factors need to be be assessed before initiating therapy. These factors include demographics, virology, host allelic variation of interleukin-28B and interferon-λ4, laboratory values and histological features [26-29]. A treatment-naïve patient with favorable predictive factors probably does not need a triple therapy but can be effectively treated with a standard antiviral therapy consisting of Peg-IFN-α and RBV.

## Conclusion

This study confirms results that have already been found in other meta-analyses, i.e., that the addition of TVR increases the likelihood of achieving a SVR in both treatment-naïve and -experienced patients. In addition, the present report suggests that the SVR achieved in treatment-experienced patients with long TVR-RGT is comparable with 48 weeks TVR-FLT with a lower risk of SAE and concurs with the rationale presented by the FDA that led to the approval of RGT in treatment-experienced patients.

Furthermore, a short, 24 week TVR-FLT regimen was not found to be inferior to a longer treatment regimen which implies that the additional 24 weeks of treatment with Peg-IFN-α and RBV does not increase the chance of achieving SVR in patients without an eRVR. It may therefore be viable to compare a shorter 24-weeks FLT regimen to a RGT treatment regimen to reduce the cost and consequences of possibly futile additional Peg-IFN-α/RBV treatment in patients without eRVR.

## Competing interests

The authors declared that they have no competing interests.

## Authors’ contributions

AA formulated the study concept, ADG designed the study, AA and ADG retrieved data from all articles independently. AA and ADG analyzed the research quality, interpreted data and wrote the manuscript. SC critically revised the manuscript for important intellectual content. All authors read and approved the final manuscript.

## Pre-publication history

The pre-publication history for this paper can be accessed here:

http://www.biomedcentral.com/1471-230X/13/148/prepub
